# Wnt Signaling Upregulates Teneurin-3 Expression via Canonical and Non-canonical Wnt Pathway Crosstalk

**DOI:** 10.3389/fnins.2019.00505

**Published:** 2019-05-17

**Authors:** Sussy Bastías-Candia, Milka Martínez, Juan M. Zolezzi, Nibaldo C. Inestrosa

**Affiliations:** ^1^Basal Center for Aging and Regeneration, Facultad de Ciencias Biológicas, Pontificia Universidad Católica de Chile, Santiago, Chile; ^2^Center of Excellence of Biomedicine of Magallanes, Universidad de Magallanes, Punta Arenas, Chile; ^3^School of Psychiatry, Centre for Healthy Brain Ageing, Faculty of Medicine, University of New South Wales, Sydney, NSW, Australia

**Keywords:** teneurin-3, Wnt signaling, Wnt3a, Wnt5a, neuronal development, C59

## Abstract

Teneurins (Tens) are a highly conserved family of proteins necessary for cell-cell adhesion. Tens can be cleaved, and some of their proteolytic products, such as the teneurin c-terminal associated-peptide (TCAP) and the intracellular domain (ICD), have been demonstrated to be biologically active. Although Tens are considered critical for central nervous system development, they have also been demonstrated to play important roles in adult tissues, suggesting a potential link between their deregulation and various pathological processes, including neurodegeneration and cancer. However, knowledge regarding how Ten expression is modulated is almost absent. Relevantly, the functions of Tens resemble several of the effects of canonical and non-canonical Wnt pathway activation, including the effects of the Wnt pathways on neuronal development and function as well as their pivotal roles during carcinogenesis. Accordingly, in this initial study, we decided to evaluate whether Wnt signaling can modulate the expression of Tens. Remarkably, in the present work, we used a specific inhibitor of porcupine, the key enzyme for Wnt ligand secretion, to not only demonstrate the involvement of Wnt signaling in regulating Ten-3 expression for the first time but also reveal that Wnt3a, a canonical Wnt ligand, increases the expression of Ten-3 through a mechanism dependent on the secretion and activity of the non-canonical ligand Wnt5a. Although our work raises several new questions, our findings seem to demonstrate the upregulation of Ten-3 by Wnt signaling and also suggest that Ten-3 modulation is possible because of crosstalk between the canonical and non-canonical Wnt pathways.

## Introduction

Teneurins (Tens; e.g., Ten-m/ODZ) are part of a conserved family of type II transmembrane proteins that are highly relevant during embryogenesis and have functions related to the proper development of the central nervous system (CNS), specifically neuronal matching and neuronal circuitry patterning (Baumgartner and Chiquet-Ehrismann, 1993; Baumgartner et al., 1994; Levine et al., 1994; Kenzelmann et al., 2007; Tucker et al., 2007; Young and Leamey, 2009; Hong et al., 2012). Ten proteins, which in vertebrates include four members (1 to 4), exhibit functions in several processes, including cell adhesion, cytoskeleton interaction, and calcium binding (Tucker and Chiquet-Ehrismann, 2006; Tucker et al., 2007). Cell adhesion and neuronal matching are dependent on Ten dimerization at cysteine residues located in the extracellular domain (Feng et al., 2002; Rubin et al., 2002; Beckmann et al., 2013). On the other hand, filopodium formation, synaptogenesis, and axonal growth and guidance also depend on the interaction of Tens with CAP/Ponsin (Nunes et al., 2005; Mosca et al., 2012), a key regulatory protein in actin polymerization (Zhang et al., 2013). Additionally, Tens can be cleaved, leading to the release of the intracellular domain (ICD), which can translocate to the nucleus and act as a transcriptional regulator (Bagutti et al., 2003; Nunes et al., 2005). Similarly, the extracellular domain of Tens can also be cleaved, leading to the release of the teneurin c-terminal-associated peptide (TCAP), which has been found to demonstrate interesting neuroactive properties (Wang et al., 2005; Tan et al., 2011). Accordingly, deregulated Tens expression during embryogenesis leads to severe alterations, including impaired binocular vision (Leamey et al., 2007; Dharmaratne et al., 2012; Young et al., 2013), microphthalmia, visual defects (Aldahmesh et al., 2012) and impaired hippocampal neuronal networking (Berns et al., 2018).

Although these observations have been restricted to the CNS, Tens are expressed in several adult tissues, suggesting that these proteins might be related to normal physiology as well as to chronic degenerative processes observed in fully developed tissues. Although information about this latter issue is scarce, altered expression of Tens has been reported in several types of cancer, including breast, ovarian, liver, and nervous system cancers (Molenaar et al., 2012; Ziegler et al., 2012a, b). Furthermore, it has recently been proposed that the expression levels of Ten-2, Ten-3, and Ten-4 might have interesting prognostic value in some types of cancer, such as ovarian cancer and neuroblastoma (Molenaar et al., 2012). On the other hand, the critical roles demonstrated by Tens in the establishment of the neuronal circuitry, especially in the hippocampal region, and the neuroactive properties of the TCAP make Tens highly interesting candidates for evaluation in the context of neurodegenerative disorders such as Alzheimer’s disease, where synaptic loss, neuronal cell death and hippocampal circuitry failure are part of the pathophysiological process (Selkoe and Hardy, 2016).

Although Ten expression has been suggested to be tightly regulated during embryogenesis by a self-regulated mechanism that depends on the overlap of Ten functions and on interactions with additional cell adhesion molecules, such as neurexin and neuroligin (Ben-Zur et al., 2000; Zhou et al., 2003; Kinel-Tahan et al., 2007; Mosca, 2015), our knowledge of the mechanisms able to modulate Ten expression, including crosstalk with known signaling pathways, is limited. Such limitation compromises our understanding of the involvement of these proteins in different cellular processes and the potential roles of these proteins as pharmacological targets for various pathological conditions.

Importantly, the activities of Tens share relevant similarities with the effects of the Wnt signaling pathway, a critical molecular pathway for CNS development and function (Bastías-Candia et al., 2015; Mosca, 2015). Moreover, several of the known functions of Tens and Wnt seem to suggest direct crosstalk between these two elements. In this regard, Wnt signaling, which can be divided into the canonical Wnt/β-catenin pathway and the non-canonical Wnt/planar cell polarity (PCP) and Wnt/Ca^++^ pathways, has been demonstrated to be critical for dendritic arborization, axonal elongation, maintenance of the synaptic architecture, and neurogenesis in the adult brain (Inestrosa and Arenas, 2010; Varela-Nallar and Inestrosa, 2013). However, the Wnt signaling pathway also constitutes a fundamental growth control pathway, and its alteration has been linked with several pathological conditions ranging from abnormal development/function of different biological systems to cancer development (Koo et al., 2015; Nusse and Clevers, 2017). Indeed, the Wnt pathway plays a critical role in carcinogenesis and is considered one of the most significant molecular pathways associated with the malignant transformation leading to tumorigenesis (Polakis, 2012).

Accordingly, given the Tens-Zic2 relationship, the presence of calcium-sensitive motifs within the structures of Tens and the already well-defined functions of Tens and the Wnt pathway, we hypothesized that there is direct communication between these pathways, which could be of significance in the context of chronic degenerative processes (Bastías-Candia et al., 2015). Thus, in the present work, we tested this hypothesis and evaluated whether Wnt signaling can modulate the expression of Ten-3, a representative Ten whose expression has been demonstrated to be necessary for CNS and hippocampal network development as well as for neuroblastoma tumorigenesis. After performing *in silico* analysis to corroborate the presence of binding motifs for TCF/Lef (the conserved canonical Wnt signaling transcription factor) in the *TEN-3* gene promoter, we used C59, a highly specific porcupine inhibitor, to ablate the Wnt signal (Hofmann, 2000; Herr et al., 2012; Ho and Keller, 2015; Koo et al., 2015; Nusse and Clevers, 2017). Interestingly, we observed that both the Wnt3a (canonical) and Wnt5a (non-canonical) ligands were able to increase basal expression of Ten-3 mRNA by up to 81 and 247%, respectively. Moreover, we observed that the Wnt3a-mediated increase in Ten-3 was dependent on the release of the ligand Wnt5a, suggesting a central role for the non-canonical pathway in Ten-3 expression. Altogether, our findings not only support Wnt-mediated modulation of Ten-3 expression but also suggest a more complex mechanism of regulation involving direct and necessary crosstalk between the canonical and non-canonical Wnt pathways. Although preliminary, our work constitutes the very first report of Wnt-Ten crosstalk and will initiate a very interesting field of research given the potential implications of such communication in the context of cell physiology and certain pathophysiological processes, including cancer and neurodegenerative disorders.

## Materials and Methods

### Identification of TCF/Lef Consensus Binding Sites on Genes of Interest

To identify potential TCF/Lef binding sites, an *in silico* analysis was carried out. Genomic sequences of the human *TEN-3* and *WNT5A* gene promoters were screened for putative DNA binding motifs using the JASPAR database with an 80% relative score threshold.

### Cell Culture and Treatments

The neuroblastoma cell line SH-SY5Y was purchased from Sigma-Aldrich (St. Louis, MO, United States) and was handled as recommended by the supplier. Briefly, cells were grown in DMEM supplemented with 10% fetal bovine serum and a 1% penicillin and streptomycin solution. The cells were allowed to reach 70% confluence prior to subculture. The cells were passaged at least eight times and were seeded onto 96-well and 12-well plates to carry out all the experiments. Additionally, some cells were seeded onto 12 mm coverslips for immunofluorescence assessment of Ten-3 expression. For experimentation, the SH-SY5Y cells were treated for 24 h with recombinant Wnt3a (150 ng/ml), Wnt5a (150 ng/ml) and Wnt7a (150 ng/ml) (R&D systems, Minneapolis, MN, United States) alone or in the presence of C59 (Tocris, Minneapolis, MN, United States).

### C59 Cytotoxicity Assay and Wnt Secretion Blockade

SH-SY5Y cells were seeded onto 96-well plates and treated with 1, 10, 100, or 200 μM C59. After 24 h in culture, cytotoxicity was evaluated using an MTT assay. Briefly, the cells were treated with different concentrations of C59 for 24 h. At the end of treatment, the cells were washed with 1× PBS, and 100 μl of fresh 1× PBS was added to each well. Then, 10 μl of thiazolyl blue tetrazolium bromide (MTT, 0.45 mg/ml) was added to each well and incubated for 3 h at 37°C. After the incubation with MTT, DMSO was added as a solubilization solution to dissolve the formazan crystals. The absorbance was measured in an iMark microplate reader (Bio-Rad, Hercules, CA, United States) at 570 nm.

Once we established the non-cytotoxic concentrations of C59, we evaluated the inhibition of Wnt ligand secretion. To do this, we used the trichloroacetic acid (TCA) protein precipitation method to assess Wnt3a levels in the extracellular medium. Briefly, after cells were treated for 24 h with 1 and 10 μM C59, the culture medium was replaced, and 100% TCA solution was added in a 1:4 (TCA:sample) ratio. The samples were incubated at 4°C for 10 min and centrifuged at 14,000 rpm for 5 min. The resulting pellet was washed twice in cold acetone and dried at 95°C for 5 min. Total protein was loaded and resolved by SDS-PAGE using a 10% polyacrylamide gel, and the separated proteins were transferred to a PVDF membrane. The membrane was incubated with a rabbit anti-Wnt3a antibody (overnight, 4°C, 1:1000, ab28472, Abcam, Cambridge, MA, United States) and an HRP-conjugated secondary antibody (1 h, room temperature, 1:5000, cat. no. 31460, Thermo Scientific, Waltham, MA, United States), and Clarity Western ECL Substrate (Bio-Rad) was used for the chemiluminescence reaction. Chemiluminescence was detected using a ChemiDoc-It 515 Imager (UVP, Upland, CA, United States).

### Immunofluorescence Staining

After treatment, SH-SY5Y cells seeded on coverslips were fixed with a 4% paraformaldehyde/sucrose solution, permeabilized with a 0.1% PBS/TWEEN 20 solution and blocked using a 1% PBS/bovine serum albumin (BSA) solution. Then, the cells were incubated with a Ten-3 (ICD) primary antibody (1:50, ab205507, Abcam) overnight. Secondary antibody (Alexa Fluor 488, 1:1000, Abcam) incubation was performed for 1 h at 37°C. Phalloidin (Alexa Fluor 568 Phalloidin, 1:50, Thermo Fisher Scientific) and TO-PRO-3 (Alexa Fluor 647 TO-PRO-3, 1:50, Thermo Fisher Scientific) antibodies were used to stain actin and nuclei, respectively. Images were captured using a Zeiss LSM5 Pascal confocal microscope (Zeiss, Oberkochen, Germany). Image analysis was carried out using ImageJ software.

### Quantitative PCR (qPCR)

RNA extraction was performed using the TRIzol (Invitrogen, Carlsbad, CA, United States) method according to the manufacturer’s instructions. The extracted RNA was quantified in a microplate spectrophotometer (BioTek, Winooski, VT, United States), and 500 ng of RNA was used for reverse transcription with Superscript IV (Invitrogen). Primers for qPCR were custom designed and synthesized by Integrated DNA Technologies (IDT, Skokie, IL, United States). The mRNA levels of Ten-3, Wnt5a and Cyclin D1 were determined using the ^ΔΔ^Ct method with GAPDH as the housekeeping control. The primer sequences used to perform qPCR were as follows: GAPDH, 5′-AGACAGCCGCATCTTCTTGT-3′ (forward) and 5′-CTTGC CGTGGGTAGAGTCAT-3′ (reverse); Ten-3, 5′-CGGGTACCCA CACAGAAGTC-3′ (forward) and 5′-GCCTTAGGGTAAAATT CTGTCCTTG-3′ (reverse); Wnt5a, 5′-AACTGGCGGGACTTT CTCAA-3′ (forward) and 5′-GTCTCTCGGCTGCCTATTTG-3′ (reverse); and Cyclin D1, 5′-GACCCCGCACGATTTCATTG-3′ (forward) and 5′- AAGTTGTTGGGGCTCCTCAG-3′ (reverse).

### Statistical Analysis

The data are presented as the mean ± SEM. The data were transferred to Excel spreadsheets after collection. Statistical analysis was carried out using Prism 6 software v.6.0h (GraphPad, La Jolla, CA, United States). The experiments were conducted in triplicate, and one-way ANOVA followed by Bonferroni’s *post hoc* test was applied to identify statistically significant differences. Significance was set at *p* < 0.05 (p* = 0.05; p** = 0.01; p*** = 0.001).

## Results

### The Human Ten-3 Promoter Region Possesses Several TCF/Lef Binding Motifs, Suggesting Canonical Wnt-Dependent Modulation

**FIGURE 1:**
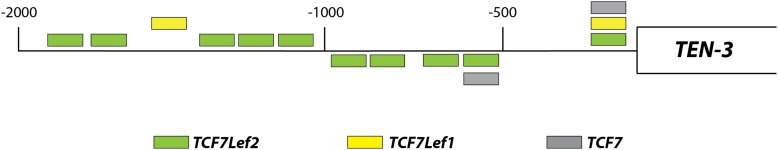
Relative locations of TCF/Lef binding motifs in the promoter region of *TEN-3*. Up to 14 potential TCF/Lef binding motifs were found 2 kb upstream of the *TEN-3* transcription start site. Three of these motifs were located in the most proximal 500 bp region, four were located in the second 500 bp region, and seven were located in the last 1 kb region. The box represents the TEN-3 gene, and the line represents the 2 kb upstream of the gene. Green: TCF7Lef2; yellow: TCF7Lef1; and gray: TCF7.

Using an *in silico* approach, we assessed the potential regulation of Ten-3 expression by canonical Wnt signaling. A total of 14 TCF/Lef binding motifs were found up to 2 kb upstream of the transcription start site of Ten-3. These motifs were located homogeneously throughout the 2 kb region, with three motifs in the most proximal 500 bp region, four in the following 500 bp region, and seven in the last 1 kb region (Figure 1). This initial observation strongly suggested that the canonical Wnt/β-catenin pathway might regulate the expression of Ten-3. Accordingly, we tested *in vitro* whether Wnt3a, a well-known canonical Wnt ligand, could induce the expression of Ten-3.

### C59, a Specific Porcupine Inhibitor, Blocks Wnt3a Secretion in SH-SY5Y Cells

**FIGURE 2:**
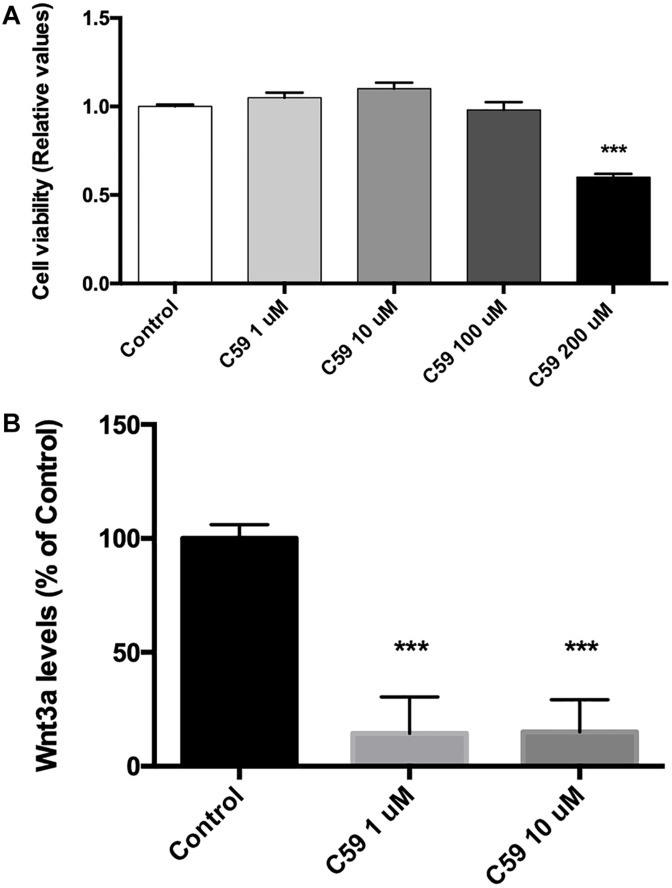
C59 cytotoxicity and inhibitory concentration for the secretion of Wnt ligands. **(A)** Cytotoxicity was evaluated through assessment of mitochondrial functionality with an MTT assay. Treatment with C59 for 24 h at concentrations over 100 μM reduced SH-SY5Y vitality, causing a mortality rate of up to 40% at the 200 μM concentration (0.6 ± 0.011, ***p < 0.001). Although a slight decrease in SH-SY5Y vitality was observed at 100 μM, this difference was not significant. **(B)** The inhibitory concentration of C59 was estimated based on the results of the cytotoxicity assay. Thus, 1 μM and 10 μM C59 were tested. After 24 h of C59 treatment, the levels of the ligand Wnt3a, a representative Wnt ligand, were assessed in the culture medium using the trichloroacetic acid precipitation method. The concentration of Wnt3a was decreased by up to 85% under treatment conditions compared with control conditions (1 μM: 14.49 ± 9.18, ***p < 0.001; 10 μM: 14.92 ± 8.17, ***p < 0.001).

Prior to evaluating the effects of Wnt3a on Ten-3 expression, we used the compound C59, a well-known and highly selective porcupine inhibitor, to ablate the basal Wnt signal. To do so, we treated SH-SY5Y cells with different concentrations of C59, and we evaluated both the cytotoxicity of C59 and the inhibition of the secretion of the ligand Wnt3a. After 24 h of treatment, it was evident that C59 affected cell survival when a dose greater than 100 μM was administered, inducing up to 40% mortality at 200 μM (0.6 ± 0.011, p***) (Figure 2A). Based on this result, we evaluated whether 1 μM and 10 μM C59 effectively prevented Wnt3a secretion. As expected, both concentrations almost completely abolished Wnt3a in the extracellular medium, reducing the levels of the ligand by up to 85% (1 μM: 14.49 ± 9.18, p***; 10 μM: 14.92 ± 8.17, p***) (Figure 2B).

### Wnt3a Increases Ten-3 Signal and mRNA Levels in SH-SY5Y Cells

**FIGURE 3:**
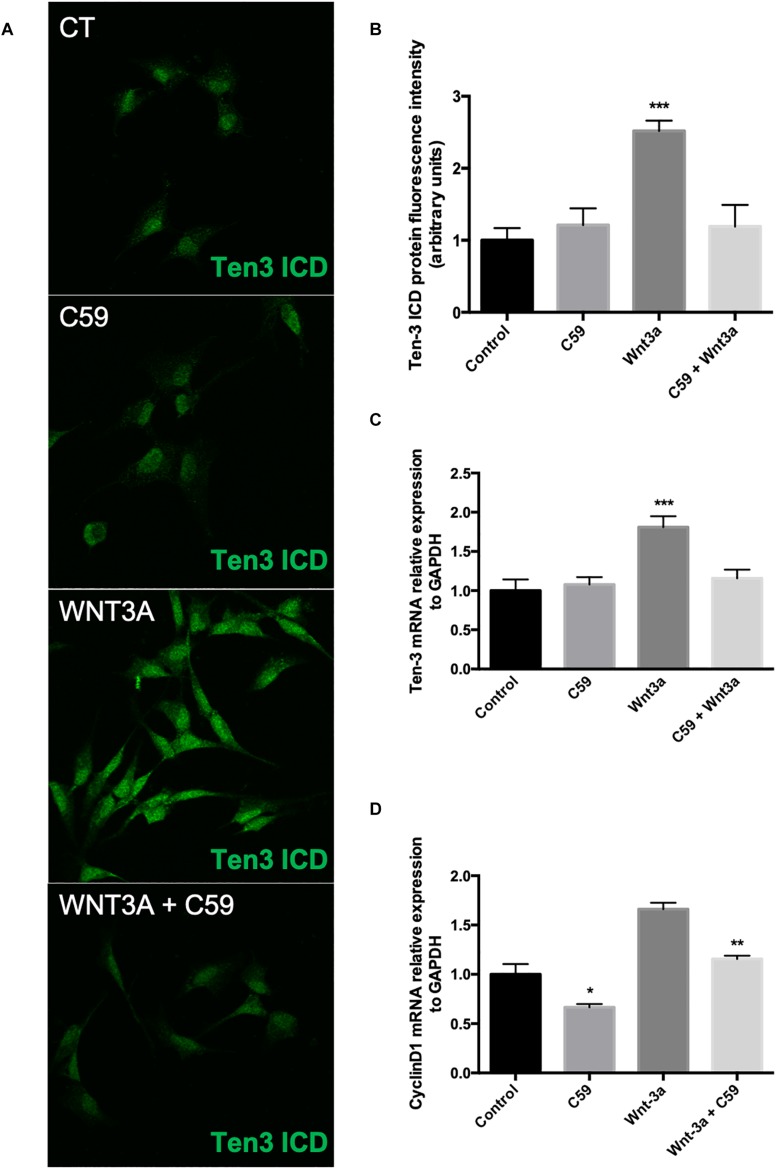
Exogenous Wnt3a increases the expression of Teneurin-3 in SH-SY5Y cells. **(A)** Representative photomicrographs of SH-SY5Y cells treated with Wnt3a (150 ng/ml), C59 (10 μM) and Wnt3a+C59. **(B)** Immunofluorescence (IF) quantification revealed a 1.5-fold increase in Ten-3 intracellular domain (ICD) signal intensity after treatment with the ligand Wnt3a (2.52 ± 0.064, ***p < 0.001). The effect was completely lost when Wnt3a was used in combination with C59. **(C)** Quantification of mRNA levels showed that the levels of Ten-3 mRNA increased when SH-SY5Y cells were treated with Wnt3a (1.81 ± 0.07, ***p < 0.001 compared to control levels). As observed in the IF results, the combination of Wnt3a and C59 abolished the effect of Wnt3a on Ten-3 mRNA levels. **(D)** C59 treatment significantly decreased the levels of cyclin D1 mRNA (0.66 ± 0.032, *p < 0.05 compared to control levels). However, exogenous Wnt3a prevented this decrease and recovered cyclin D1 mRNA to the levels in control cells, which were significantly different than those in C59-treated cells (1.16 ± 0.032, **p < 0.01).

After determining the concentration of C59 to be used, we evaluated the effects of the ligand Wnt3a on the expression of Ten-3. After 24 h of treatment with 150 ng/ml recombinant Wnt3a, the treated cells showed 1.5-fold higher Ten-3 (ICD) signal levels than the control cells (2.52 ± 0.064, p***) (Figures 3A,B). On the other hand, the cells treated with 10 μM C59 exhibited a small reduction in the Ten-3 (ICD) signal compared to control cells (Figures 3A,B). However, co-incubation of cells with Wnt3a and C59 completely prevented the Wnt3a-induced increase in the Ten-3 (ICD) signal (Figures 3A,B). Interestingly, through analysis of mRNA levels, we confirmed that Wnt3a increases the expression of Ten-3 mRNA (1.81 ± 0.07, p***) but that this increase is abolished in the presence of C59 (Figure 3C). To eliminate the possibility of an issue with the effectiveness of the Wnt3a stimulus, we determined the mRNA expression levels of Cyclin D1, a well-established canonical Wnt target gene, in the same samples. C59-treated cells exhibited a significant decrease in cyclin D1 mRNA compared with control cells (0.66 ± 0.032, p*); however, the cyclin D1 levels recovered and slightly increased when Wnt3a was added (Wnt3a+C59 compared with C59; 1.16 ± 0.032, p**) (Figure 3D). Considering the specificity of the inhibitor C59, these results suggest that Wnt3a modulates Ten-3 expression by affecting the secretion of a secondary Wnt ligand. Moreover, considering that the canonical ligand was not able to counterbalance the effects of C59 on Ten-3 mRNA levels, we inferred that non-canonical Wnt signaling might be part of the molecular mechanism involved in the upregulation of Ten-3 expression.

### Wnt3a Increases the mRNA Levels of the Ligand Wnt5a in SH-SY5Y Cells

**FIGURE 4:**
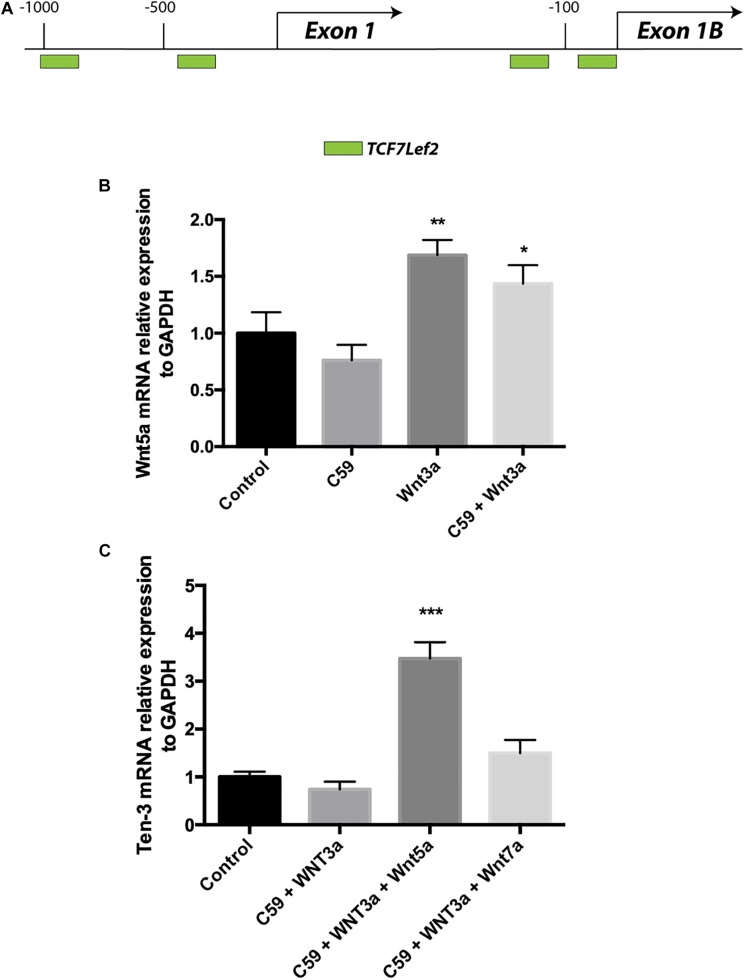
Wnt3a induces Wnt5a transcription, and Wnt5a dramatically increases Teneurin-3 mRNA levels. **(A)** Graphic representation of the promoter regions of the WNT5A gene. Through *in silico* analysis, 2 potential TCF/Lef binding motifs were found at each of the two promoter regions described for the WNT5A gene. At promoter A, two binding sites were located at –1000 and –400 bp upstream of the exon 1 transcription start site. On the other hand, at promoter B, the two binding sites were located in the first 150 bp upstream of the exon 2 transcription start site. Green: TCF7Lef2. **(B)** Wnt5a mRNA levels were significantly increased in both the Wnt3a and Wnt3a+C59 groups compared with the control groups (1.69 ± 0.077, **p < 0.01 and 1.44 ± 0.094, *p < 0.05; respectively). **(C)** Cells treated with 150 ng/ml Wnt5a had 247% higher Ten-3 mRNA levels than control cells (3.47 ± 0.199, ***p < 0.001).

Accordingly, we conducted a second *in silico* evaluation to screen for TCF/Lef binding sites, this time in the promoter region of the WNT5A gene, a representative non-canonical Wnt ligand. Interestingly, the *in silico* analysis showed that even though Wnt5a exists in two isoforms and thus has two promoter regions, TCF/Lef binding sites are present in both regions (Figure 4A). After verifying the sites and using the same samples to evaluate the Ten-3 mRNA levels, we assessed the expression of the non-canonical ligand Wnt5a. Interestingly, Wnt5a mRNA levels were significantly higher in Wnt3a- and Wnt3a+C59-treated cells compared to control cells (1.69 ± 0.077, p**, and 1.44 ± 0.094, p*, respectively) (Figure 4B). This finding further suggests that the canonical ligand Wnt3a might induce the expression of Ten-3 but in a manner linked to the secretion and activity of the non-canonical ligand Wnt5a.

### Wnt5a Dramatically Increases Ten-3 mRNA Levels in the Presence of the Inhibitor C59

To confirm the above finding, we proceeded to evaluate cells treated with Wnt3a+C59, this time adding Wnt5a or Wnt7a recombinant ligands. In this case, Wnt7a was included as an additional control for the Wnt canonical pathway. Remarkably, when Wnt5a was added to the Wnt3a+C59 group, the expression level of Ten-3 mRNA increased to 247% of the level under control conditions (3.47 ± 0.199, p***). In contrast, the Wnt7a ligand was not able to replicate this result, indicating that the effect of Wnt3a on Ten-3 mRNA levels was Wnt5a-dependent (Figure 4C).

## Discussion

In recent years, the role of Tens in the developing nervous system has been well documented, demonstrating that appropriate expression of this family of proteins is mandatory for neuronal development and neuronal network formation. Recent data have also pointed out a significant role for Tens in the context of adult tissue biology (Ziegler et al., 2012b). Indeed, Tens and its proteolytic products, such as TCAP and ICD, have been linked with important effects on the adult nervous system, including the management of addiction and anxiety (Kupferschmidt et al., 2011; Tan et al., 2011; Erb et al., 2014). Similarly, other studies have shown that aberrant Ten expression is associated with tumor development and malignancy, suggesting that specific Tens can be used as valuable prognostic cancer biomarkers (Molenaar et al., 2012; Ziegler et al., 2012a, 2012b). Together, these findings strongly suggest that Tens play relevant roles in the maintenance and physiology of fully developed tissues outside of the CNS. Surprisingly, despite the depicted importance of these proteins and the wide range of biological effects that these proteins seem to mediate, little information is available about the regulatory mechanisms of their expression, including the modulatory effects of well-known cellular signaling pathways. As mentioned in the introductory section, the similarities between Ten functions and those described for Wnt signaling, mainly in the context of the CNS, prompted us to evaluate our former hypothesis and determine whether activation of the Wnt pathway could modulate Ten expression.

As a starting point, we evaluated the promoter region of *TEN-3* for the presence of TCF binding motifs. Remarkably, the *in silico* analysis revealed 14 potential binding sites for the TCF family of transcription factors, with ten of these sites corresponding to TCF7/Lef2 (Figure 1). In this regard, although several TCF family members (1 to 4) have been shown to exert opposing regulatory effects when bound to β-catenin, TCF7/Lef2 has been systematically shown to increase the expression of its target genes (Nakano et al., 2010; Cadigan and Waterman, 2012; Ramakrishnan and Cadigan, 2017). Our initial screening further indicated the potential involvement of the Wnt signaling pathway, particularly the canonical branch, in the modulation of Ten-3 expression.

Based on this initial finding, we proceeded to evaluate the involvement of the ligand Wnt3a, a main representative of the canonical branch of the Wnt pathway, on the expression levels of Ten-3. Considering that most of the information regarding Tens has been reported for the nervous system and for cancer, we decided to use the SH-SY5Y cell line, a neuronal model and a representative neuroblastoma-derived cell line. Moreover, to properly evaluate the effects of exogenous Wnt3a on Ten-3 expression levels, we used C59, a specific inhibitor of porcupine; porcupine is an exclusive regulatory enzyme of Wnt ligand palmitoylation, which is mandatory for Wnt ligand secretion and bioactivity (Herr et al., 2012; Proffitt et al., 2013; Wend et al., 2013; Ho and Keller, 2015; Koo et al., 2015; Bernatik et al., 2017; Nigmatullina et al., 2017; Nusse and Clevers, 2017). Indeed, under our experimental conditions, C59 almost completely abolished Wnt ligand secretion without affecting cell survival, at least at the 1 μM and 10 μM concentrations (Figure 2). Notably, even though we observed that exogenous Wnt3a significantly increased the expression levels of Ten-3 at both the mRNA and protein levels, Wnt3a was unable to induce Ten-3 expression when C59 was present (Figures 3A,B). To corroborate the effectiveness of the exogenous Wnt3a treatment, we evaluated the mRNA levels of Cyclin D1, a conserved canonical Wnt pathway target gene, in the same samples. Interestingly, we observed that Wnt3a was able to prevent the reduction in Cyclin D1 expression caused by C59, confirming that Wnt3a induced the effects of C59. Based on these findings, we hypothesized that Wnt3a was able to induce Ten-3 expression but that this effect involved the secretion and activity of a secondary Wnt ligand. Moreover, considering that the canonical ligand was unable to counteract the effects of C59 on Ten-3 mRNA levels, as observed with Cyclin D1, we inferred that non-canonical Wnt signaling may be part of the molecular mechanism involved in the upregulation of Ten-3 expression.

Accordingly, we conducted an additional *in silico* analysis to screen for TCF binding motifs, this time in the promoter region of Wnt5a, a well-studied representative non-canonical Wnt ligand that has been suggested as a potential target of the canonical Wnt pathway (Hödar et al., 2010). Our analysis showed that both promoter regions of the *WNT5a* gene contain two TCF7/Lef2 binding motifs, suggesting that both Wnt5a isoforms are subject to canonical Wnt modulation. Indeed, when we evaluated the levels of Wnt5a mRNA in the samples previously exposed to Wnt3a and Wnt3a+C59, we observed significant increases in Wnt5a mRNA expression of up to 70 and 45%, respectively (Figures 4A,B). This finding suggested that Wnt3a probably induced the increased expression of Wnt5a but that because of the inhibitory effect of C59, the Wnt5a ligand was not palmitoylated, affecting its secretion and activity. Thus, we investigated whether exogenous Wnt5a could overcome the inhibitory effects of C59. Remarkably, when we introduced Wnt5a into the system, we observed a dramatic increase in the levels of Ten-3 mRNA, which reached values up to 247% of those in control cells. In addition, to establish the specific role of Wnt5a in these effects, we used Wnt7a as a secondary canonical Wnt ligand. In this case, no significant increase in Ten-3 mRNA levels was observed (Figure 4C).

**FIGURE 5:**
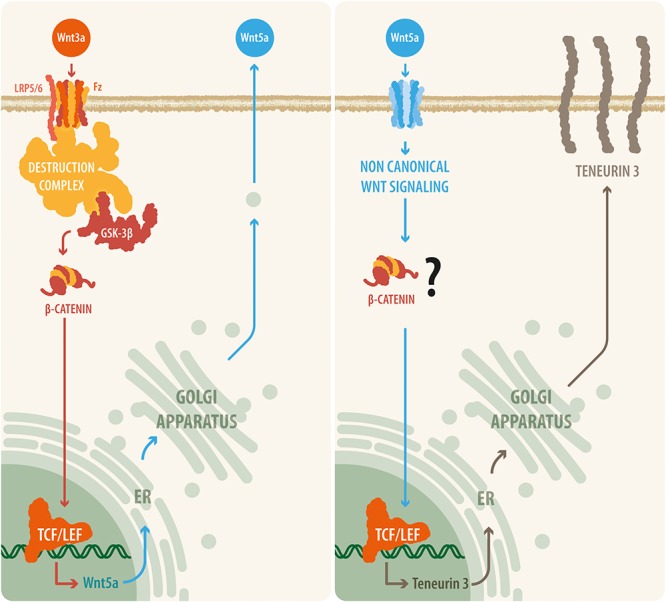
A proposed model relating canonical and non-canonical Wnt signaling with teneurin-3 expression. Our results suggest that upon Wnt3a activation, the canonical Wnt pathway is triggered, leading to the disassembly of the β-catenin destruction complex and to the accumulation of β-catenin within the cell. β-Catenin can then translocate to the nucleus, where it binds to the TCF/Lef transcription factor and thus causes the synthesis and release of the non-canonical ligand Wnt5a. Then, Wnt5a is released by the cell and is able to act in an autocrine or a paracrine manner, initiating transcription of the *TEN-3* gene. In addition, the observation that several TCF7/Lef2 motifs are present in the promoter region of the *TEN-3* gene and that Wnt5a can signal through β-catenin via ADP-ribosylation factor 6 (ARF6) activity seem to further support this suggested cooperative mechanism between the canonical and non-canonical Wnt pathways.

Together, our results demonstrate that Ten-3 expression can be regulated by Wnt signaling. Moreover, they suggest that even though the non-canonical branch can induce Ten-3 expression independently through the ligand Wnt5a, the canonical branch requires a cooperative mechanism involving both the canonical and non-canonical Wnt pathways. Similar cooperation between canonical signals and non-canonical signals, specifically Wnt5a, has been reported previously (Schulte et al., 2005; Andersson et al., 2013). Moreover, as has been stated for the canonical Wnt pathway, Wnt5a has been found to be related to neuronal development, axonal guidance, neuronal branching, and organ innervation (Kumawat and Gosens, 2016). Remarkably, it has been shown that Wnt5a not only activates the non-canonical pathway but also can activate canonical signaling through activation of the GTPase ADP-ribosylation factor 6 (ARF6) after FZD4-LRP6 binding, allowing β-catenin-related gene transcription (Grossmann et al., 2013). Considering the various TCF7/Lef2 binding sites in the promoter region of Ten-3, it is possible that the protein expression of Ten-3 is mediated by crosstalk between the canonical and non-canonical Wnt pathways, specifically between the ligands Wnt3a and Wnt5a, with Wnt5a acting as the final effector of this modulatory mechanism through β-catenin/TCF7/Lef2 signaling (Figure 5). Furthermore, because we used SH-SY5Y cells and observed increases in Ten-3 mRNA expression, our results are in agreement with the recent report of Szemes et al. (2018), which indicates that in neuroblastoma, Wnt3a acts as a differentiation factor (making the cancer less malignant). Considering that Ten-3 expression has also been linked to reduced neuroblastoma malignancy, we hypothesize that Wnt3a/Wnt5a-mediated Ten-3 expression might be associated with the Wnt3a/Wnt5a context-dependent protective effects against neuroblastoma (Grossmann et al., 2013).

## Conclusion

The Ten family has emerged as a fascinating family of proteins because of the critical roles Tens play in the development of the CNS. Importantly, Tens have also been demonstrated to be critical for the maintenance and physiological functioning of adult tissues. However, information regarding the regulatory mechanisms of Tens is completely absent. Moreover, considering that Tens have been linked to important pathological processes, the relevance of novel regulatory mechanisms and the roles of significant cellular pathways in the modulation of Tens should not be overlooked (Südhof, 2018). In this work, we report not only the very first mechanism of the regulation of Ten-3 expression but also that this mechanism involves interplay between the canonical Wnt ligand Wnt3a and the non-canonical Wnt ligand Wnt5a. We believe that these two findings are of great relevance to understanding the roles of Tens, particularly Ten-3, under physiological conditions and to understanding how Ten proteins might interact with molecular pathways that define cell fate in specific contexts, including in different pathophysiological processes such as cancer and neurodegeneration. Therefore, we believe that our work demonstrates, for the first time, the Wnt-mediated upregulation of Ten-3 through a novel Wnt3a-Wnt5a complementary signal representing a coupling of the canonical and non-canonical Wnt pathways.

We must highlight that this work constitutes an initial approach to elucidate the involvement of Wnt signaling in the regulation of Tens expression. In this sense, although our results demonstrate a Wnt-Ten interaction, new questions have emerged, including those regarding the mechanisms underlying Wnt5a-induced Ten-3 expression given the dual action of Wnt5a as a canonical and non-canonical activator of Wnt signaling. Further studies will be necessary to properly address these questions, but we believe that our work offers an interesting starting point from which to develop new research aimed at establishing the molecular mechanisms involved in Tens expression.

## Author Contributions

SB-C and MM conducted the experiments and revised the final manuscript. JZ, SB-C, and NI designed the experiments and wrote the manuscript.

## Conflict of Interest Statement

The authors declare that the research was conducted in the absence of any commercial or financial relationships that could be construed as a potential conflict of interest.
